# Perioperative Music Implementation in Bariatric Patient Care: An Interventional Study

**DOI:** 10.7759/cureus.78281

**Published:** 2025-01-31

**Authors:** Kayleigh van Dam, Victor Fu, Geert Verkoulen, Pieter Broos, Evelien de Witte, Jan Willem Greve, Evert-Jan Boerma

**Affiliations:** 1 Surgery, Zuyderland Medical Center, Heerlen, NLD; 2 Surgery, Institute of Nutrition and Translational Research in Metabolism, Maastricht University, Maastricht, NLD; 3 Bariatric Care, Dutch Obesity Clinic South, Heerlen, NLD

**Keywords:** bariatric metabolic surgery, music, nausea, pain, roux-en-y gastric bypass

## Abstract

Background:* *Perioperative music can positively affect postoperative pain, opioid requirement, and anxiety. These effects are even present when music is played solely during general anesthesia. This study assesses the effect of implementing perioperative music as standard patient care in elective bariatric metabolic surgery (BMS).

Methods:This prospective study compared the preimplementation (control) and postimplementation (intervention) groups between March and October 2023. The control group did not receive music, while the intervention group received patient-selected music using headphones and a tablet. Music was provided as standard during anesthesia. Only patients undergoing elective BMS (primary ring augmented Roux-en-Y gastric bypass) were included. The primary outcome was postoperative pain on a 10-point scale (numeric rating scale, NRS) on the first postoperative day. The secondary outcomes included postoperative nausea, patient satisfaction, and opioid and antiemetic requirements.

Results*: *In the control group, 66 patients were included, while 65 were included in the intervention group. Median NRS scores were 5 in both groups, showing no significant difference (p = 0.325). Medication use (analgesics and antiemetics) was similar in both groups. Patient satisfaction scores were high at 8 (8-9) and 9 (8-10), respectively, with no significant difference (p = 0.137). In the intervention group, most patients (86%) want to listen to music during subsequent surgical procedures.

Conclusion:Implementing perioperative music in BMS did not significantly reduce postoperative pain or postoperative medication use. As the bariatric perioperative tract is already well-received without music, it poses a challenge for detecting notable improvements. However, the strong patient preference for music during future surgeries emphasizes the positive perception of music in perioperative care.

## Introduction

Perioperative music has demonstrated various therapeutic benefits for surgical patients, including improvement in postoperative pain and anxiety, reductions in intraoperative sedation and postoperative opioid requirements, and attenuation in physiological stress responses to surgery [1-3]. These effects have also been observed even when music is played exclusively during general anesthesia [4]. Given its lack of adverse effects, perioperative music is considered an appealing nonpharmacological intervention, particularly valuable in the context of the ongoing opioid crisis [2]. Furthermore, the implementation of music therapy aligns well with modern fast-track perioperative protocols designed to achieve faster patient recovery and hospital discharges [5]. Even though previous studies, including meta-analyses, have shown the benefits, including high patient satisfaction and minimal time investment, perioperative music is not yet part of standard surgical patient care [2,6,7].

The underlying mechanisms through which music influences the perioperative experience are increasingly understood. For instance, neuroimaging studies indicate that music stimulates the release of endogenous opioids and dopamine [3,8]. Music reduces sympathetic nervous system activation, resulting in lower heart rate, blood pressure, and cortisol levels, which are markers of the stress response to surgery [3,9]. By reducing the physiological stress response, music may help create a more favorable recovery environment and, thus, reduce the need for pharmacological interventions.

The use of perioperative music has been described in a few types of surgeries, especially abdominal and orthopedic surgery [1-3,7]. These studies consistently demonstrate reductions in postoperative pain, anxiety, and medication requirements. However, its specific impact on bariatric metabolic surgery (BMS) remains unexplored. As same-day discharge becomes increasingly common in BMS, it is important to understand how music can benefit these patients [8]. Nevertheless, introducing any intervention involves challenges that require implementation research to identify and address contextual factors and barriers at the patient, physician, and organizational levels [9]. These implementation studies differ from randomized controlled trials by focusing on reproducibility in everyday clinical settings. Therefore, the aim of this study was to evaluate the effect of implementing perioperative music on patients undergoing elective BMS.

## Materials and methods

Study design

The study was a prospective, single-center implementation study comparing the preimplementation (control) and postimplementation (intervention) groups. Patients undergoing an elective primary ring augmented Roux-en-Y Gastric Bypass (RYGB) were included. The control group did not receive music at the surgical complex, while the intervention group was exposed to music during the surgical procedure. The sample size was calculated to include 130 patients in total based on previous studies reporting an effect size of at least 0.5 for postoperative pain. The required sample size was determined using two-tailed testing with α = 0.05 and β = 0.80. For this prospective data study, local approval was given by the local ethics committee in accordance with the ethical standards laid down in the 2013 Declaration of Helsinki.

Patient selection

Patients of >18 years who underwent a primary RYGB procedure between March and October 2023 at the Zuyderland Medical Center were included. Sufficient knowledge of the Dutch language and the ability to complete the questionnaire was required. After obtaining written consent, patients were assigned to either the control or intervention group. The patients were chronologically assigned to either group, as the control group was filled first, followed by the intervention group.

Music intervention

The control group did not receive music. Therefore, they did not get a tablet and headphones. These patients were only required to complete a questionnaire on the first postoperative day, which could be filled in on their own mobile devices via e-mail. The intervention group was introduced to the music intervention on the morning of surgery. Patients were given a tablet and headphones, along with access to a music streaming platform (Spotify, Stockholm, Sweden) to select their preferred music. Patients could select their own music, as previous studies showed the same effectiveness of self-selected music compared to predetermined music [10,11]. Patients received the tablet upon arrival at the admission ward and instructions for use. The patients could start listening to music in the preanesthesia room if they wanted. Music was played for all patients in this group during the surgical procedure. After surgery, patients could choose to continue listening to music in the recovery room and at the ward. The nursing staff collected the tablets at the end of the day, typically around 5 PM. The following morning, the pre- and postimplementation group patients received the questionnaire (Appendix 1) via e-mail.

Data collection

All patients were prospectively included in the database. The baseline data included age, gender, height, weight, and BMI. Perioperative information regarding hospital admission and complications was also collected. The primary outcome was a postoperative pain score on a 10-point scale on the first postoperative day. The pain was assessed using the numeric rating scale (NRS), collected through a postoperative questionnaire (Appendix 1). The secondary outcomes included postoperative nausea, opioid and antiemetic requirements, patient satisfaction, and duration of hospital admission. The opioid requirement was converted into morphine milligram equivalents (MME), whereby, for instance, oxycodone has a 1.5 conversion rate (1 mg oxycodone = 1.5 MME). The postoperative nausea was assessed through the postoperative questionnaire using a score ranging from 0 to 2. The usage of opioids and antiemetics was collected by screening the medication overview in the electronic patient files. The actual doses administered are analyzed, rather than the simply prescribed medication.

Statistical analysis

Statistical analysis was performed using the SPSS Statistic for Windows, version 29.0 (IBM Corp., Armonk, NY). Categorical variables were presented as frequencies with percentages. Continuous variables were presented as mean ± standard deviation for normal distributed variables and median and interquartile range for a skewed distribution. Differences between the groups were tested using either Student's t-test or the Mann-Whitney U test. A p value of <0.05 was considered statistically significant. Missing data were reported as such.

## Results

A total of 131 patients were analyzed, of whom 66 were included in the control group and 65 in the intervention group. The preoperative demographic data at screening for RYGB are summarized in Table 1 and were similar for both groups. The control group had a mean age of 42 years (±10.6), and 56/66 (84.8%) were female. The intervention group had a mean age of 43.2 years (±12.1), and 49/65 (75.4%) were female. The median preoperative BMI was also comparable with a BMI of 40.9 (39.4-44.7) in the control group and a BMI of 42.2 (39.9-45.9) in the intervention group.

**Table 1:** Baseline characteristics Data are presented as mean ± standard deviation, median (IQR), or n (%) BMI: body mass index; IQR: interquartile range

Baseline characteristics	Control group (n = 66)	Intervention group (n = 65)
Age (years)	42 ± 10.6	43.2 ± 12.1
Gender		
Male	10 (15.2)	16 (24.6)
Female	56 (84.8)	49 (75.4)
Weight at screening (kg)	118 (108.2-126.1)	123.8 (108.8-139.6)
BMI at screening (kg/m^2^)	40.9 (39.4-44.7)	42.2 (39.9-45.9)

Patient-reported and questionnaire-related outcomes

Of the 131 patients, 117 (89.3%) completed the custom-made questionnaires regarding pain, nausea, and satisfaction. The questionnaire was supposed to be filled in on the first postoperative day, a target achieved by 70.9% of the respondents. The remaining 29.1% completed the questionnaire on subsequent days. The distribution of completion on day 1 was similar in both groups.

**Figure 1:**
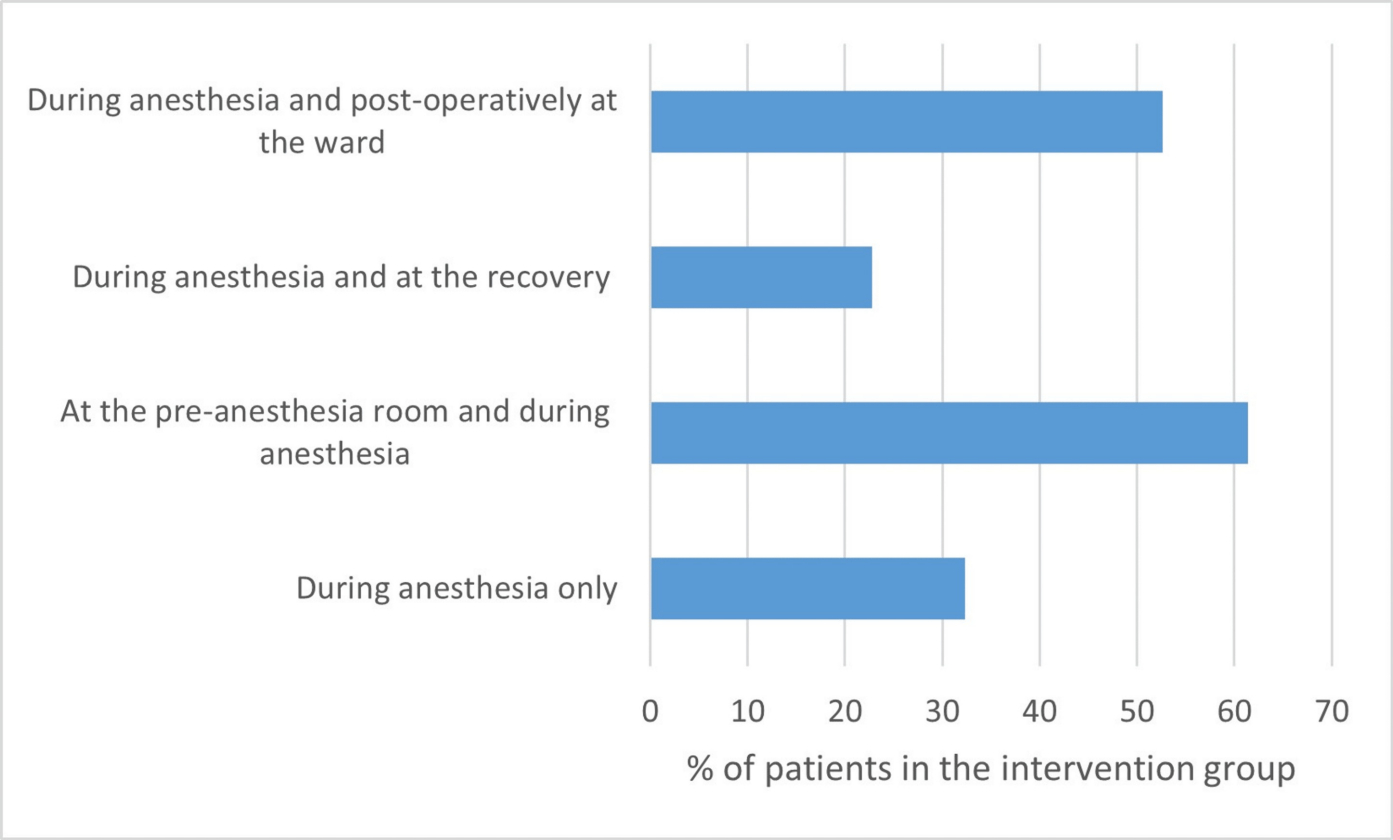
Distribution of locations where patients listened to music

In the control group, 60 patients (90.9%) did not listen to their own music at any point during their hospital stay. The remaining six patients only listened to music postoperatively in the surgical ward. In the intervention group, all patients were exposed to music during anesthesia. Patients had the opportunity to listen to music at several other places during the clinical stay, namely in the preanesthesia room, in the recovery, and in the ward (Figure 1). A total of 21 patients (32.3%) solely listened to music during anesthesia, while the remaining patients also listened to music in at least one of the other locations. Most patients listened to music directly before surgery in the preanesthesia room (61.4%). Following the procedure, 22.8% of the patients listened to music in the recovery room, and 52.6% listened to music in the surgical ward.

As shown in Table 2, the median pain score on a scale of 0-10 was 5 (3-7) in the control and 5 (3-6) in the intervention group. Statistical analysis revealed no significant differences between these groups (p = 0.325). Similarly, the median nausea score was 1 vs. 0 (0-1), with no significant difference (p = 0.366). Regarding satisfaction, the median patient score on a 10-point scale was 8 (8-9) in the control group and 9 (8-10) in the intervention group, with no significant difference (p = 0.137). For subsequent surgical procedures, most patients indicated they would want to listen to music, with 78.6% in the control group and 86% in the intervention group. For the control group, this response reflected their neutral preferences as they had not experienced the music intervention. For the intervention group, this response was based on their actual experience with music during surgery.

**Table 2:** Patient-reported scores on pain, nausea, and satisfaction Data are presented as median (IQR) or n (%) Pain on a 10-point Likert scale, nausea on a three-point Likert scale, and satisfaction on a 10-point Likert scale. The p value was calculated with the Mann-Whitney U test ^*^n represents only patients who filled in the questionnaire

Questionnaire outcomes	Control group (n = 60)^*^	Intervention group (n = 57)^*^	p value
Questionnaire completed on day 1	42 (70)	41 (71.9)	-
Pain score (0-10)	5 (3-7)	5 (3-6)	0.325
Nausea score (0-2)	1 (0-1)	0 (0-1)	0.366
Satisfaction score (0-10)	8 (8-9)	9 (8-10)	0.137

Medication requirement

Table 3 presents the postoperative analgesic and antiemetic medication requirements for patients in the control group compared to the intervention group. The mean opioid requirement, measured in MME, was 4.15 (±9) in the control group and 2.5 (±6.2) in the intervention group, with no significant difference (p = 0.394). The majority of patients in both groups required no opioids postoperatively (84.8% vs. 80%). The proportion of patients requiring 15 MME was similar between groups, with 13.6% in the control group and 12.3% in the intervention group. A slightly higher proportion of patients in the control group required 30 MME, with 1.5% vs. 7.7% in the intervention group, although this difference was not statistically significant (p = 0.394).

**Table 3:** Postoperative analgesic and antiemetic medication requirement Data are presented as mean ± standard deviation or n (%) Antiemetic requirement was measured in milligrams of ondansetron. The p value was calculated using the Mann-Whitney U test MME: morphine milligram equivalents

Medication outcomes	Control group (n = 66)	Intervention group (n = 65)	p value
Opioid requirement (MME)	2.5 (±6.2)	4.15 (±9)	0.394
Distribution opioid requirement (MME)			
0	56 (84.8)	52 (80)	0.394
15	9 (13.6)	8 (12.3)	
30	1 (1.5)	5 (7.7)	
Antiemetic requirement (mg)	1.11 (±3)	1 (±2.5)	0.868
Distribution antiemetic requirement (mg)			
0	54 (81.8)	54 (83.1)	0.876
4	8 (12.1)	7 (10.8)	
8	3 (4.5)	2 (3.1)	
12	1 (1.5)	1 (1.5)	
16	0 (0)	1 (1.5)	

The mean antiemetic requirement following surgery, measured in milligrams of ondansetron, was comparable between the control and intervention groups (1.11 ± 3 vs. 1 ± 2.5, p = 0.868). The distribution of antiemetic use was similar between groups. The majority of patients in both groups required no antiemetics postoperatively, with 81.8% in the control group and 83.1% in the intervention group. A small proportion of patients required higher doses, with similar distributions across the dose categories with no statistical significance (p = 0.876).

## Discussion

This study evaluated the impact of perioperative music on postoperative outcomes in patients undergoing BMS. Postoperative outcomes included pain, nausea, medication use, and patient satisfaction. While previous studies [1-4] have shown benefits of music interventions in other types of surgery, such as reduced pain and anxiety, this study found no statistically significant differences.

Previous studies have demonstrated varying results, with several meta-analyses demonstrating reduced pain scores and opioid requirements across various procedures, including abdominal, oncologic, and orthopedic surgeries and coronary artery bypass grafts [1,2]. However, a recent randomized controlled trial by Fu et al. showed no differences in pain scores between the music and control groups (1.8 vs. 2.2) for gastrointestinal procedures [11].

In our study, both groups had a mean NRS of 5, which is indicative of moderate pain. The threshold for severe pain in laparoscopic bariatric surgery is defined as >7 [12]. Comparable to our results, the study by Chen et al., which evaluated the effects of music in knee replacement procedures, found no differences in moderate postoperative pain scores (4.9 vs. 5.1) [13]. However, Chen et al. did show a reduced preoperative respiratory rate in the music group. Another study evaluated the effect of music in laparoscopic hysterectomy and only showed a significant benefit in the preoperative time period with a reduction of preoperative anxiety [14]. Regarding the pain and nausea scores, no differences between the music and control groups were found. Notably, both of the previously mentioned studies had a specific preoperative music phase as part of their intervention [13,14]. Our study did not incorporate any standardized preoperative music listening as the primary focus was on music during the procedure and anesthesia itself.

The long-term benefits are not studied extensively. Only the study evaluating music implementation during laparoscopic cholecystectomy suggested potential long-term benefits [15]. The immediate outcomes were similar to other studies, with no differences in pain scores on the first postoperative day. However, after a seven-day period, there were notable differences observed in fatigue and pain scores, as the music group reported lower fatigue and pain levels [15]. This might suggest an effect that extends beyond the immediate recovery period. Our study did not assess long-term effects but focused on outcomes during the clinical stay, which was often limited to one day.

Our study's findings are specific to patients undergoing primary BMS, which could be a key factor influencing the results. The BMS care path includes highly optimized perioperative care according to the Enhanced Recovery after Bariatric Surgery (ERABS) protocol [5,16]. The patients undergoing primary BMS have received preoperative education and counseling. The ERABS includes tailored anesthetic techniques and specific analgesia to minimize opioid use. Implementing these protocols likely minimized baseline pain and nausea scores, even without additional interventions such as music therapy.

Limitations

This study has some limitations that need to be acknowledged. First, due to the single-center design, these findings may not be generalizable to other institutions with different perioperative protocols. Additionally, the variability in the timing of the music was not controlled, which could influence the outcomes. Second, the headphones provided to patients were not noise-canceling devices. This might have reduced the overall effectiveness of the intervention due to external noises or distractions.

Furthermore, BMS has a unique context compared to other types of surgeries as the patients have typically received thorough preoperative education on the perioperative care path. This may reduce baseline levels of anxiety and stress. The primary Roux-en-Y Gastric bypass is standardized in terms of surgical technique, perioperative care, and patient selection. Perhaps with revisional or conversional BMS, which are often more complex or variable, a more suitable context for evaluating the benefits of music therapy can be achieved. Especially the conversional procedures often have increased technical complexity and longer operative times, which, in turn, can lead to higher levels of preoperative anxiety, postoperative pain, and overall stress. Under these circumstances, the effect of music therapy might be more noticeable.

## Conclusions

The implementation of perioperative music did not result in significant changes in pain, nausea, or medication requirements among patients undergoing elective BMS. As the bariatric perioperative tract is already highly optimized, it is challenging to detect notable improvements. While music does not appear to be an essential addition to this care path, the high patient satisfaction scores and the strong preference for music during future procedures emphasize its value as a patient-centered intervention. Future studies should further evaluate the effect of music during other surgical procedures or conversional BMS to identify patient groups that may benefit the most and to optimize the timing of the therapy.

## Appendices

Appendix 1

*Translated Q* *uestionnaire Preimplementation Group*

1. Check the box that best describes how you are currently experiencing your pain, with 0 representing no pain and 10 representing the worst possible pain.

 No pain                                                                                                        Worst possible pain

      0          1           2           3           4           5           6           7           8           9           10

     □         □          □         □           □          □          □          □          □          □          □

2. Check the box that best describes how you are currently experiencing your nausea, with 0 representing no nausea, 1 average nausea, and 2 severe nausea.

        *   No nausea                   Average nausea           Severe nausea*

                   0                                      1                                  2

                  □                                     □                                 □

3. Which music genre do you prefer (select up to three options)? □   Blues                                             □   Country □   Hip Hop                                        □   Jazz □   Classical                                        □   Metal □   Dutch folk music                          □   Pop □   Rhythm and blues (R&B)             □   Rock □   I don't know                                 □   Other, namely:

4. How satisfied are you with the provided patient care, where 0 represents very dissatisfied and 10 represents very satisfied?

Very dissatisfied                                                                                                      Very satisfied

      0          1           2           3           4           5           6           7           8           9           10

     □         □          □         □           □          □          □          □          □          □          □

5. If you were to undergo surgery again, would you want to listen to music again?

□   Yes □   No

6. Did you listen to music on your own phone/tablet during admission? □   Yes □   No

Translated Questionnaire Postimplementation Group

1. Check the box that best describes how you are currently experiencing your pain, with 0 representing no pain and 10 representing the worst possible pain.

 No pain                                                                                                        Worst possible pain

      0          1           2           3           4           5           6           7           8           9           10

     □         □          □         □           □          □          □          □          □          □          □

2. Check the box that best describes how you are currently experiencing your nausea, with 0 representing no nausea, 1 average nausea, and 2 severe nausea.

   *        No nausea                   Average nausea           Severe nausea*

                   0                                      1                                  2

                  □                                     □                                 □

3. Which music genre do you prefer (select up to 3 options)? □   Blues                                             □   Country □   Hip Hop                                        □   Jazz □   Classical                                        □   Metal □   Dutch folk music                          □   Pop □   Rhythm and blues (R&B)             □   Rock □   I don't know                                 □   Other, namely:

4. How satisfied are you with the music equipment, where 0 represents very dissatisfied and 10 represents very satisfied?

Very dissatisfied                                                                                                      Very satisfied

      0          1           2           3           4           5           6           7           8           9           10

     □         □          □         □           □          □          □          □          □          □          □

5. How satisfied are you with the provided patient care, where 0 represents very dissatisfied and 10 represents very satisfied?

Very dissatisfied                                                                                                      Very satisfied

      0          1           2           3           4           5           6           7           8           9           10

     □         □          □         □           □          □          □          □          □          □          □

6. If you were to undergo surgery again, would you want to listen to music again?

□   Yes □   No

7. Where did you listen to music during your stay? (Multiple answers possible)

□   Operating room complex (before surgery) □   During surgery □   Recovery □   Ward
